# Distinct Responses of Arabidopsis Telomeres and Transposable Elements to Zebularine Exposure

**DOI:** 10.3390/ijms22010468

**Published:** 2021-01-05

**Authors:** Klára Konečná, Pavla Polanská Sováková, Karin Anteková, Jiří Fajkus, Miloslava Fojtová

**Affiliations:** 1 Mendel Centre for Plant Genomics and Proteomics, Central European Institute for Technology (CEITEC), Masaryk University, Kamenice 5, CZ-62500 Brno, Czech Republic; klara.konecna@ceitec.muni.cz (K.K.); pavla.sovakova@ceitec.muni.cz (P.P.S.); karin.antekova@gmail.com (K.A.); jiri.fajkus@ceitec.muni.cz (J.F.); 2 Laboratory of Functional Genomics and Proteomics, National Centre for Biomolecular Research, Faculty of Science, Masaryk University, Kotlářská 2, CZ-61137 Brno, Czech Republic; 3 Institute of Biophysics, Academy of Sciences of the Czech Republic, Královopolská 135, CZ-61265 Brno, Czech Republic

**Keywords:** *Arabidopsis thaliana*, epigenetics, cytosine methylation, telomeres, transposable elements, zebularine

## Abstract

Involvement of epigenetic mechanisms in the regulation of telomeres and transposable elements (TEs), genomic regions with the protective and potentially detrimental function, respectively, has been frequently studied. Here, we analyzed telomere lengths in *Arabidopsis thaliana* plants of Columbia, Landsberg erecta and Wassilevskija ecotypes exposed repeatedly to the hypomethylation drug zebularine during germination. Shorter telomeres were detected in plants growing from seedlings germinated in the presence of zebularine with a progression in telomeric phenotype across generations, relatively high inter-individual variability, and diverse responses among ecotypes. Interestingly, the extent of telomere shortening in zebularine Columbia and Wassilevskija plants corresponded to the transcriptional activation of TEs, suggesting a correlated response of these genomic elements to the zebularine treatment. Changes in lengths of telomeres and levels of TE transcripts in leaves were not always correlated with a hypomethylation of cytosines located in these regions, indicating a cytosine methylation-independent level of their regulation. These observations, including differences among ecotypes together with distinct dynamics of the reversal of the disruption of telomere homeostasis and TEs transcriptional activation, reflect a complex involvement of epigenetic processes in the regulation of crucial genomic regions. Our results further demonstrate the ability of plant cells to cope with these changes without a critical loss of the genome stability.

## 1. Introduction

Telomeres are functionally and structurally distinct chromatin domains at the ends of linear eukaryotic chromosomes. At the DNA level, they are formed by conserved minisatellite repetitive sequences, for example, TTTAGGG in most plants [1] and TTAGGG in vertebrates [2]. Telomeres distinguish natural chromosomal ends from double-strand DNA breaks, protect them against fusion and activities of repair complexes, and act as buffer zones against the replicative loss of DNA coding sequences (reviewed in [3]).

As chromatin structures, telomeres are natural targets for epigenetic modifications, methylation of cytosines in DNA, and posttranslational modifications of histones. In both these categories, specific features of plant telomeric chromatin have been described. Plant DNA methyltransferases are able to methylate cytosines in all sequence contexts, including non-symmetrical CHH (H = A, T, C) motifs [4]. Thus, cytosines in the C-rich telomeric strand can be methylated and, correspondingly, methylated cytosines in *Arabidopsis thaliana* telomeric repeats can be detected by high-throughput bisulfite sequencing. The inner cytosine within the CCCTAAA sequence unit is the most frequently methylated, while outer cytosines are methylated to significantly lower extents [5]. This pattern, at least in the proximal part of the telomere, has been confirmed by subsequent studies [6,7], but questioned by others [8,9].

Transposable elements (TEs), mobile genomic elements that are involved in processes of genome evolution through mutagenic transposition, are highly abundant in plant genomes (reviewed in [10]). *A. thaliana* has played a crucial role in TE research, because using this model plant, the nature and control of TEs have been described including the significant role of epigenetic processes. DNA methylation and modifications of histones contribute to the silencing of TEs at the level of transcription, and RNA interference is involved in their posttranscriptional regulation [11,12,13,14]. Interestingly, although transcription of TEs is elevated in mutants with a loss of function of DDM1 protein, which is crucial for the maintenance of methylation of cytosines in all sequence contexts, transposition activity is detected in a relatively small number of TEs [12,13].

Epigenetically active drugs are beneficial for functional analysis of the involvement of epigenetic mechanisms in many cellular processes. In recent studies, utilization of toxic and relatively unstable DNA hypomethylation drugs 5‑azacytidine or 5‑aza‑2′‑deoxycytidine has been replaced with another cytidine analogue, zebularine (1‑(β‑D‑ribofuranosyl)‑1,2‑dihydropyrimidine-2-one) [15]. Like for other epigenetically active cytidine analogues, incorporation of zebularine into DNA during replication and formation of the nucleoprotein adduct with DNA methyltransferase has been considered [16]. This mechanism of the zebularine hypomethylation activity has been challenged because the rate of the zebularine incorporation into genomic DNA was significantly lower as compared with 5‑azacytidine [17] and, in Arabidopsis, zebularine incorporation was even below the detection limit [18]. Specific zebularine-induced DNA damage, related probably to the formation of zebularine-DNA methyltransferase adducts and repaired by intermolecular homologous recombination (HR), has been reported as an effect that dominated changes in DNA methylation in Arabidopsis [18]. In spite of the fact that the mechanism of zebularine hypomethylation activity has not been fully elucidated, the impact of this drug at the level of methylated cytosines is evident. A reduction in DNA methylation due to the zebularine treatment has been reported in Arabidopsis and *Medicago sativa* seedlings [19] and tobacco BY-2 cells [20]; the hypomethylation capacity of zebularine during germination of Arabidopsis seeds was comparable to that of 5-azacytidine [21] or even slightly stronger [19].

The effect of zebularine has also been analyzed with respect to TEs and telomeres. Transcriptome analysis revealed TEs to be the most upregulated class in Arabidopsis following zebularine treatment [21]. Regarding telomeres, the length of telomeres of tobacco BY-2 cells cultured in a medium supplemented with zebularine were maintained [20], while distinct telomere shortening was observed in leaves of Arabidopsis plants of the Columbia ecotype grown from seedlings exposed to zebularine during germination [6].

Here, we analyzed three consecutive generations of plants of three Arabidopsis ecotypes growing from seeds exposed, at each generation, to the zebularine during germination, and correlated modulation of the telomere phenotype with the level of transcriptional activation of selected TEs. Although significantly shortened telomeres and high levels of TEs transcripts were detected in leaves of zebularine plants, the maintenance of these changes was not dependent on hypomethylation of cytosines in these genomic regions.

## 2. Results

### 2.1. Zebularine-Induced Gradual Shortening of Telomeres in Arabidopsis Plants of Columbia-0 (Col), Wassilevskija (Ws), and Landsberg erecta (Ler) Ecotypes

**Figure 1:**
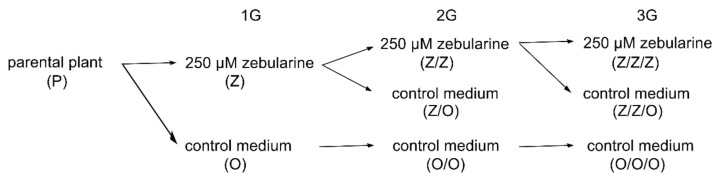
Experimental design. Seeds of *A. thaliana* of Columbia-0 (Col), Landsberg erecta (Ler), and Wassilevskija (Ws) ecotypes were germinated for 7 days on a control medium (O) or a medium supplemented with 250 µM zebularine (Z), and plants were cultivated in soil without the drug (first generation, 1G). Seeds from one parental plant (P) were germinated to limit the contribution of inter-individual variability in telomere lengths. Seeds from selected 1G plants were germinated either on a medium supplemented with zebularine (Z/Z) or on a control medium (Z/O and O/O), and plants were grown in the soil (second generation, 2G). The same protocol was followed once more to get the third generation (Z/Z/Z, Z/Z/O, and O/O/O, 3G) plants. This nomenclature is used in the following text and figures.

Building on our previous observations of telomere shortening in leaves of Arabidopsis plants of the Columbia-0 ecotype grown from seedlings germinated in the presence of hypomethylation drugs zebularine or (S)‑9‑(2,3‑dihydroxypropyl)adenine ((S)-DHPA) [6], we addressed the following two tasks: (i) to compare the extent of zebularine-induced telomere shortening in leaves of Arabidopsis plants of three ecotypes with naturally different telomere lengths [22], i.e., Columbia-0 (Col), Landsberg erecta (Ler), and Wassilevskija (Ws) and (ii) to examine the impact of the repeated exposure to the drug on telomere homeostasis. The experimental design and the nomenclature of zebularine-exposed and control seedlings and plants grown from these seedlings are depicted in Figure 1.

**Figure 2:**
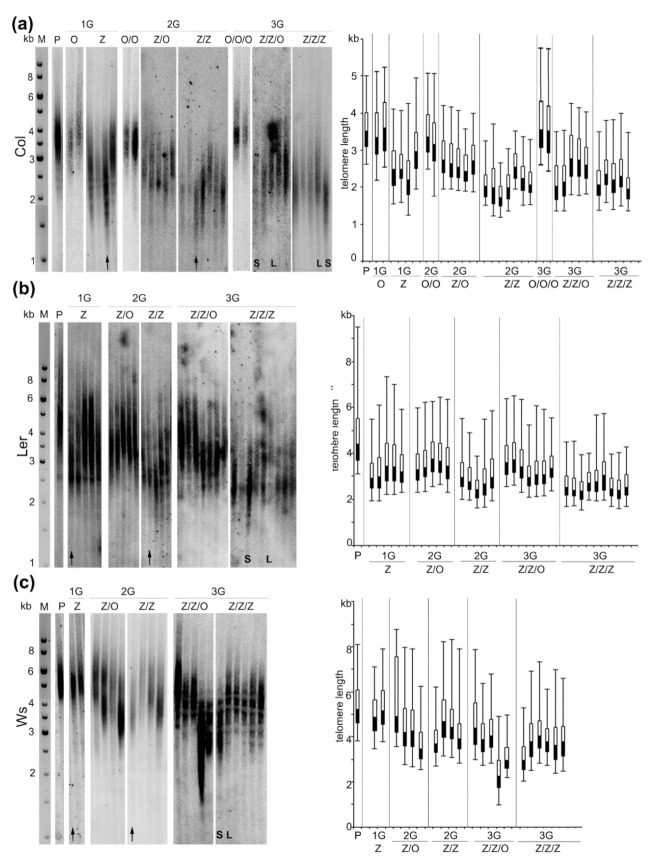
Lengths of telomeres in *A. thaliana* plants grown from seeds exposed to the zebularine during germination. (**a**) Seeds of Columbia-0 (Col); (**b**) Landsberg erecta (Ler); (**c**) Wassilewskija (Ws) ecotypes were exposed to the 250 µM zebularine during germination for three generations; in parallel, progenies of zebularine plants were germinated on a control medium (for experimental design and nomenclature of samples, see Figure 1). Leaves for analysis were collected from 8-week-old plants. DNA was extracted from 2 g of leaves, 5 µg of DNA was digested by MseI restriction endonuclease and analyzed by Southern hybridization using radioactively labeled telomeric probe. Each sample (line) corresponded to the leaf taken from one independently cultivated plant. P, parental plant; Z, first generation (1G) of plants grown from seedlings germinated in the presence of 250 µM zebularine; Z/Z, plants grown from seedlings germinated for two generations (2G) in the presence of 250 µM zebularine; Z/Z/Z, plants grown from seedlings germinated for three generations (3G) in the presence of 250 µM zebularine; Z/O, 2G plants grown from seedlings of 1G zebularine plants (Z) germinated on control medium; Z/Z/O, 3G plants grown from seedlings of 2G zebularine plants (Z/Z) germinated on a control medium. Black arrows indicate plants that were propagated to the subsequent generation. In 3G plants with distinct telomere phenotypes (S, short telomeres and L, long telomeres), the levels of transposable elements (TEs) transcripts, TE methylation and methylation of telomeric cytosines were analyzed (Figures 3–5). M, DNA fragment size marker; GeneRuler 1 kb DNA Ladder (Thermo Fisher Scientific). In the right panels, lengths of telomeres were presented using a box and whisker plot with the bottom part (black) and the top part (white) representing the lower and upper quartiles, respectively, separated by the median. The ends of whiskers represent the minimal and maximal telomere lengths reflecting the range of the hybridization signal.

Telomere shortening was observed in leaves of Col plants cultivated from seedlings germinated in the presence of zebularine (Z), with a distinct inter-individual variability (Figure 2a). In an extreme case, the extent of telomere shortening was more than 1 kb. Progenies (2G) of this plant with the shortest telomeres germinated on the control medium (Z/O) exhibited longer telomeres as compared with the parental plant but still significantly shorter than the plants without the zebularine history (O/O), in agreement with our previous report [6]. A high variability in telomere lengths was detected in zebularine 2G plants (Z/Z) with similar or slightly shorter telomeric tracts as compared with the parental Z plant. The pattern of telomere lengths in progenies of the Z/Z plant cultivated on the control medium (Z/Z/O) resembled that in the previous generation, i.e., longer telomeres than the Z/Z parent but shorter than the parental Col plants (P) and plants without the zebularine history (O/O/O). Telomeres in Z/Z/Z representatives were comparable or slightly longer as compared with the Z/Z parental plant. According to these data, the extent of zebularine‑induced telomere shortening in Col plants was limited and its progression terminated in 2G zebularine plants. As expected, the lengths of telomeres were maintained in Col plants propagated under standard conditions (O, O/O, O/O/O, Figure 2a).

In Ler Z plants, the shortening of telomeres was observed as compared with parental (P) plant (Figure 2b). In 2G and 3G, the dynamics of changes in telomere length was similar to Col plants, showing the following: (i) slightly shorter or stable telomeres in Z/Z and Z/Z/Z individuals as compared with the Z and Z/Z parental plants, respectively; (ii) maintained or elongated telomeres in plants germinated on a control medium (Z/O and Z/Z/O); and (iii) a considerable inter-individual variability.

Telomeres of Arabidopsis plants of the Ws ecotype displayed a moderate response to the zebularine treatment (Figure 2c). In the 1G Z plants, the lengths of telomeres remained at the same level as in the parental (P) plant. Individuals with shorter telomeres were detected in Z/Z and Z/Z/Z plants and, interestingly, shortened telomeres were detected in Z/O and Z/Z/O plants. Again, a relatively high variability in telomere lengths among plants within respective groups was observed, similar to the results of analyses of Col and Ler representatives.

### 2.2. Extent of Changes in Telomere Lengths Correlated with Transcriptional Activation of Transposable Elements in Col and Ws Zebularine Plants

In Arabidopsis Z/Z/Z plants of Col, Ler, and Ws ecotypes, distinctly shorter telomeres were observed as compared with the parental (P) plant. The extent of telomere shortening varied significantly among individuals (Figure 2), indicating some variability in the way in which zebularine influenced the telomere phenotype. Considering the fact that the impact of the zebularine exposure during seed germination is complex, we correlated the telomere phenotype in Z/Z/Z plants with levels of transcripts of selected TEs; levels of transcripts of these TEs were significantly higher in Arabidopsis seedlings germinated in the presence of zebularine [19,21] or 5-azacytidine [21].

**Figure 3:**
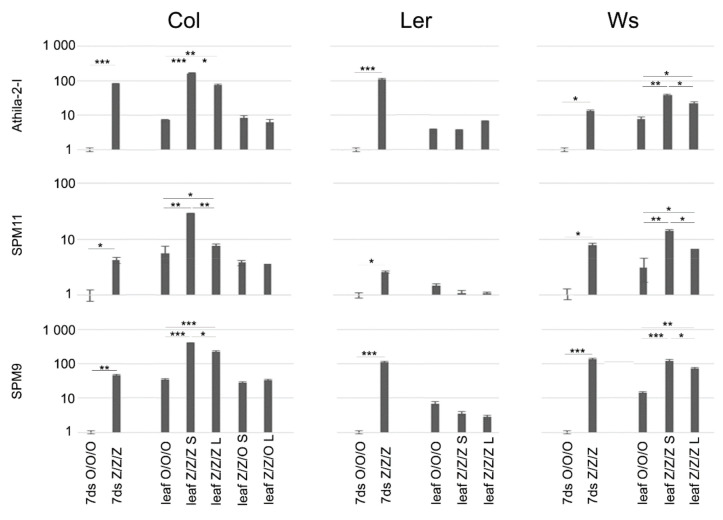
Relative levels of transcripts of selected TEs in seedlings of *A. thaliana* plants exposed repeatedly to 250 µM zebularine during germinations and in leaves of plants grown from these seedlings. RNA was extracted using the NucleoSpin RNA kit (Macherey-Nagel) from 100 mg of 7-day-old seedlings and leaves of 8-week-old plants. Relative levels of transcripts in Z/Z/Z 7-day-old seedlings (7ds) and leaves were compared to levels detected in control organs (7ds O/O/O, leaf O/O/O/, for experimental design and nomenclature of samples, see Figure 1). S, plants with short telomeres and L, plants with long telomeres (see Figure 2a). Signals of TE transcripts were related to that of the *ubiquitin10* reference gene, levels of TE transcripts in 7ds O/O/O samples were arbitrarily chosen as 1. Data were evaluated by two sample F-test and t-test. * *p* < 0.05, ** *p* < 0.01, and *** *p* < 0.001. Note the logarithmic scale at the y axis.

Relative levels of TE transcripts were analyzed in control (O/O/O) and 3G zebularine-exposed (Z/Z/Z) seedlings (i.e., organ in the direct contact with the drug), leaves of O/O/O plant, and leaves of Z/Z/Z plants with distinct telomere phenotypes (Figure 2). Compared to the control seedlings, levels of TE transcripts were significantly higher in Z/Z/Z seedlings of all three ecotypes, although the extent of transcriptional activation varied, both among ecotypes with regard to respective TE, and among TEs with regard to respective ecotype (Figure 3). Levels of TE transcripts increased in leaves of Col and Ws control plants as compared with the control seedlings, while in Ler leaves, a significant change in transcript level was detected for SPM9 transposon only. High levels of transcripts of all analyzed TEs were detected in leaves of Col and Ws Z/Z/Z plants. Thus, the exposure of seeds to zebularine during germination brought about the activation of TE transcription in seedlings and this effect persisted and was even stronger in leaves of adult plants that were no longer exposed to the drug during the growth in the soil. Interestingly, a comparison of the rate of TE transcriptional activation and telomere phenotype of Col and Ws Z/Z/Z plants (Figure 2a,c and Figure 3) indicated that significantly higher levels of TE transcripts were found in plants with shorter telomeres. These data showed different but correlated responses of individual Col and Ws plants to the zebularine treatment with respect to telomere homeostasis and TE transcriptional activation.

The analysis of TE transcripts in leaves of Col Z/Z/O plants displaying different telomere phenotypes (see Figure 2a), revealed the same or slightly lower levels of TE transcripts as detected in Col control leaves (Figure 3). Transgenerational reversal of zebularine-induced transcriptional activation of TEs was, thus, faster than that of the telomere phenotype.

The levels of TE transcripts in Ler leaves were completely different. Although transcription of TEs was activated in zebularine-exposed Ler seedlings, we did not observe any increase in transcript levels in leaves of Z/Z/Z plants with either short or long telomeres (Figure 3). Transcriptional activation of TEs detected in Ler Z/Z/Z seedlings, thus, did not last in leaves of plants that were grown from these seedlings, in contrast to Col and Ws ecotypes.

As a control, levels of transcripts of the *PAC1* gene encoding 20S proteasome subunit were analyzed. As expected, comparable levels of *PAC1* transcripts (related to levels of *ubiquitin10* transcript) were detected in all samples (Figure S1).

### 2.3. The Maintenance of the Short Telomere Phenotype and Transcriptional Activation of TEs in Leaves of Z/Z/Z Plants Are Not Tightly Related to the Loss of Methylated Cytosines in These Regions

**Figure 4:**
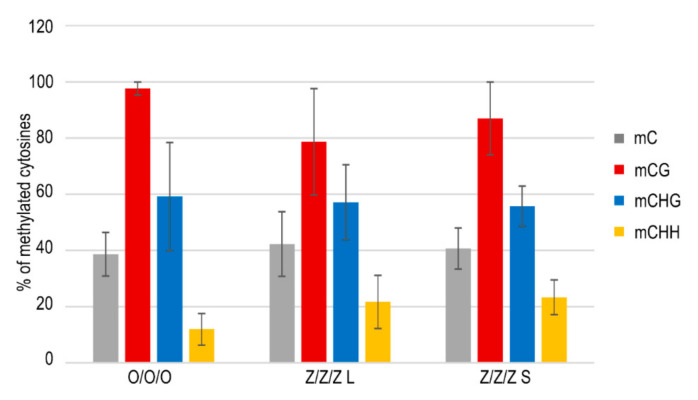
SPM11 transposon methylation analyzed by bisulfite sequencing. Methylation of 44 cytosines located in an amplified 336 bp region (6 cytosines in the CG sequence context, 14 cytosines in the CHG, 24 cytosines in the CHH, H = A, T, C) was evaluated by the CyMATE software [27]. Five to ten clones were analyzed per sample; mean values of methylated cytosines and standard deviations are presented. O/O/O, leaf of the control Col plant; Z/Z/Z L, leaf of 3G plant with long telomeres grown from seedlings exposed to zebularine during germinations; Z/Z/Z S, leaf of 3G plant with short telomeres grown from seedlings exposed to zebularine during germinations (see Figure 2a). For experimental design and nomenclature of samples, see Figure 1.

According to available data, silencing of transposons is controlled epigenetically, and genomic regions in which transposons are localized are usually rich in methylated cytosines. Therefore, DNA methylation has been considered to be an important regulatory mechanism protecting plant and vertebrate genomes from the deleterious activity of TEs [23,24]. In agreement with this hypothesis, TEs were activated in Arabidopsis mutants with affected DNA methylation [12,13,25] and a similar mechanism was proposed for zebularine-induced transcriptional activation of TEs. Nevertheless, no significant differences in levels of methylated cytosines within the SPM11 transposon region were detected among leaves of the control Col plants (O/O/O) and Z/Z/Z plants with long or short telomeres, except for some decrease in the CG methylation in the Z/Z/Z plant with long telomeres (Figure 4). Similar results were obtained previously for MULE2 and LINE1–4 transposable elements; elevated transcript levels of these TEs were detected in true leaves of Arabidopsis plants grown from seedlings exposed to zebularine during germination without the change of the level of methylated cytosines [26]. Thus, and rather surprisingly, zebularine-induced transcriptional activation of TEs was not correlated with their current hypomethylation.

**Figure 5:**
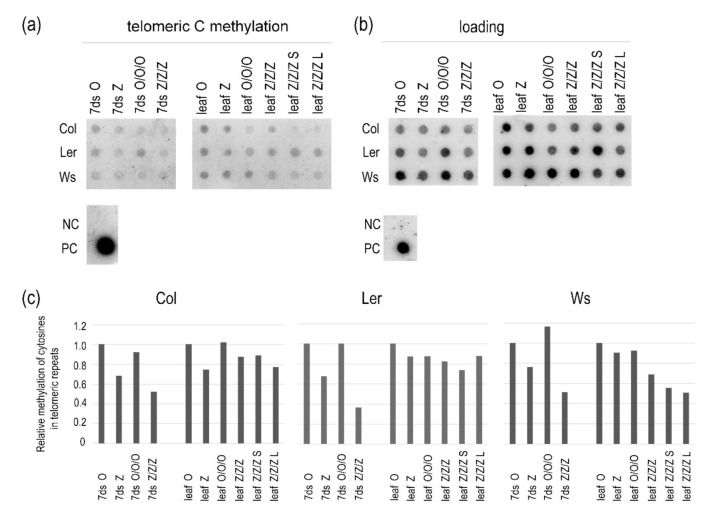
Relative levels of methylated cytosines in telomeric repeats in the first and third generations of 7-day-old seedlings (7ds) of *A. thaliana* plants germinated on control medium or exposed to 250 µM zebularine, and in leaves of plants grown from these seedlings in soil. Mixed samples were analyzed; DNAs were isolated from 1 g of seedlings cultivated on three Petri dishes and 2 g of leaves of three plants grown from these seedlings, except of individual 3G zebularine plants with short and long telomeres (leaf Z/Z/Z S, leaf Z/Z/Z L). For bisulfite conversion, DNAs were mixed at an equimolar ratio. Bisulfite-modified DNAs were hybridized with the oligonucleotide probe reflecting fraction of telomeres with methylated cytosines (**a**), and the probe complementary to the G-strand of telomeres to determine loading (**b**). The same membrane was sequentially hybridized with both probes. PC, positive control, genomic DNA from Col leaves non-converted by sodium bisulfite; NC, negative control, pUC19 plasmid DNA. (**c**) Relative methylation of cytosines in telomeric repeats. Intensities of hybridization signals in (**a**,**b**) were evaluated by the MultiGauge software (FujiFilm) and expressed as the methylation/loading ratio. Signal ratios in respective control samples of the first generation (7ds O, leaf O) were arbitrarily taken as 1. For experimental design and nomenclature of samples, see Figure 1.

Previously, we reported decreased levels of methylated telomeric cytosines in 1G seedlings of *A. thaliana* of the Col ecotype germinated in the presence of 250 µM zebularine. Interestingly, in leaves of 1G plants grown from these seedlings in soil, levels of methylated cytosines were comparable with control samples [6]. In this study, we analyzed methylation of telomeric cytosines in 1G and 3G control and zebularine seedlings and leaves of three Arabidopsis ecotypes. A decrease in the levels of methylated cytosines in telomeric repeats was observed in 1G Z seedlings and was even more pronounced in 3G Z/Z/Z seedlings (Figure 5 and Figure S2). In Col and Ler Z and Z/Z/Z plants, the levels of methylated cytosines were comparable with that in the control O/O/O sample. Thus, even considerable loss of methylated cytosines within telomeric repeats due to the repeated exposure of seeds to zebularine during germination was reversed in leaves of Col and Ler plants, despite the maintenance of the short telomere phenotype.

Different dynamics of methylation of telomeric cytosines was observed in Ws plants (Figure 5 and Figure S2). The hypomethylation of telomeric cytosines was more distinct in Z/Z/Z seedlings as compared with Col and Ler samples, and lower levels of methylated cytosines in telomeric repeats were also detected in leaves of Z/Z/Z plants with both short and long telomeres.

Importantly, relative levels of methylated cytosines were comparable between respective 1G and 3G control samples (Figure 5).

## 3. Discussion

Epigenetic mechanisms represent a powerful and complex regulatory toolbox, primarily involved in the modulation of chromatin structure and, consequently, transcription of settled gene(s). In addition, structures of regions that do not contain protein-coding genes are ordinarily determined epigenetically and changes in the combinatorial patterns of epigenetic marks influence the properties and functions of these regions. In our previous research, significant truncation of telomere tracts was detected in Arabidopsis plants of the Col ecotype exposed to the hypomethylation drug zebularine during germination [6]. Here, we considerably extended our analyses and demonstrated that repeated exposures of Col seeds to zebularine during germination induced progressive changes in telomere phenotype. However, these changes were limited, and lengths of telomeres were comparable in 2G and 3G zebularine-treated plants. The same dynamics of zebularine-induced changes in telomere lengths was observed in plants of the Ler ecotype, but in Ws zebularine plants, telomere shortening was delayed by one generation (Figure 2). Interestingly, the delayed onset of telomere shortening in response to the zebularine treatment resembled the distinctive behavior of Ws during cell culturing, although this process correlated with gradual telomere elongation. While in Col and Ler calli, telomere elongation was evident after one month of cultivation, this was delayed by four months in the Ws callus [28]. Therefore, our observations demonstrate a difference in the Ws response to the epigenetic stress, at least at the level of timing of changes in telomere lengths. The diverse outcomes of zebularine-induced changes in telomere length among Arabidopsis ecotypes of relatively close genetic backgrounds further point to a limited general validity of similar findings across plants of different species or even larger taxonomic groups.

In proposing a putative mechanism by which zebularine affects telomere lengths, its hypomethylation activity and induction of a specific type of DNA damage were considered. The short telomere phenotype and hypersensitivity to the induction of DNA double-strand breaks were previously observed in Arabidopsis *ddm1* mutants. This correlation raised the hypothesis that genome hypomethylation-induced increase in HR and stochastic deletional recombination led to telomere shortening [29]. Intermolecular HR has been reported to be a dominant mechanism for detoxification of zebularine-induced DNA damage, independently of changes in DNA methylation [18]. As this type of DNA damage is cell-cycle dependent and occurs predominantly during DNA replication [18], it may compromise the stability of late-replicating telomeres. This scenario may also explain the limited reduction in telomere lengths. Although DNA replication is slowed down due to the repair of the zebularine-induced DNA damage, telomeres are still replicated to an extent sufficient for accomplishment of their essential cellular functions. In this context, it should be noted that our study did not aim at clarifying details of the mechanism of zebularine effects in plant cells. Thus, changes in telomere maintenance and levels of TEs transcripts, described in our study as a consequence of the zebularine exposure during seed germination, are ascribed to its hypomethylation activity, in accordance with previous observations of these effects in genetically hypomethylated plants [6,12,13,29]. Nevertheless, it is not possible to exclude at least a contribution of other zebularine-induced changes in plant cells to these processes.

Transcriptional activation of TEs has been reported to be a consequence of the genome hypomethylation, including zebularine treatment [19,21]. In our experiments, levels of transcripts of selected TEs increased significantly in zebularine-exposed seedlings of all Arabidopsis ecotypes studied (Figure 3). When correlating telomere phenotype with the extent of TE transcriptional upregulation in leaves of 3G zebularine plants, a distinct interrelationship was observed in Col and Ws plants, i.e., higher levels of TE transcripts were detected in plants with shorter telomeres. In zebularine 3G Ler plants with short telomere phenotypes, levels of TE transcripts were comparable to those in control leaves. This transient transcriptional activation of TEs in zebularine-treated Ler tissues was in agreement with previously published results, where silencing of TEs was quickly re‑established in true leaves of Col and Ws plants germinated for seven days in the presence of 20 µM zebularine followed by a seven day recovery period [26]. To determine whether high levels of TE transcripts in leaves of Col and Ws zebularine 3G plants were related to a higher concentration of zebularine used in our experiments, or to repeated exposure to zebularine during germination, TE transcripts were analyzed in leaves of 1G zebularine Col plants. Levels of TE transcripts in Z leaves were comparable to those detected in Z/Z/Z leaves, i.e., significant increases were observed in both samples [30]. Thus, the persisting transcriptional activation of TEs in true leaves following zebularine exposure was related very probably to the high concentration of the drug used in our experimental setup. In our previous research dealing with the analysis of the zebularine impact on stability of telomeres in tobacco cultured cells [20] and cell culture-induced elongation of Arabidopsis telomeres [28], lower zebularine concentrations (approximately by one order of magnitude) were used. This experimental setup was adopted based on the high sensitivity of tobacco cells to the presence of hypomethylation drugs in a cultivation medium; actually, in the presence of 50 µM zebularine, BY‑2 cells were surviving only one one-week passage [20]. This may be related to the generally different composition of tobacco genome and epigenome compared to Arabidopsis (reviewed in [31]), and thus different strength of the response to the epigenome modulation by the epigenetically active drug. In our experiments, Arabidopsis seeds tolerated high zebularine concentration during seven days of germination without any obvious phenotype changes, and also plants grown from seedlings exposed to zebularine during the germination period were of the standard phenotype [32]. Nevertheless, when calli introduced from Arabidopsis seedlings were propagated for several months in the medium supplemented by zebularine [28], these were not able to survive long term exposure to high zebularine concentrations. As lengths of telomeres are used to be analyzed in leaves of adult plants, i.e., in organs that are collected several weeks following the zebularine exposure, our aim was to apply a maximal non-toxic zebularine dose to be able to observe zebularine-induced changes despite their possible instability, as the effect of zebularine on plant DNA methylation was described as transient [19].

Although the extents of zebularine-induced telomere shortening and TE transcript levels were correlated in Z/Z/Z plants of the Col ecotype, the dynamics of the reversal of these changes were different. While only partial reversal of the telomere phenotype was observed in Z/Z/O plants (Figure 2a), TE transcripts returned to control levels in both plants analyzed (Figure 3). One can speculate that the dynamics of reversal of zebularine-induced changes may correspond to the potential risk of these alterations; while telomeres shortened by 1 kb are still long enough to ensure protection of chromosomal ends, transcriptional activation of TEs represents a danger of their mobilization and deleterious insertions.

A decrease in the level of methylated cytosines has been proposed as a cause of TE transcriptional activation [12,13,25], but in our data (Figure 4), levels of methylated cytosines within the SPM11 transposon were not markedly different between Col control and Z/Z/Z plants. In agreement with these data, analysis of methylation of other types of TEs in zebularine-treated cotyledons with significantly elevated transcription, and in true leaves in which silencing of TEs was re-established, revealed modulated levels of methylated cytosines in neither of these organs, despite the mild hypomethylation of centromeric repeats [26]. Moreover, zebularine-induced dispersion of chromocenters, over a range similar to the *ddm1* mutant, was described in Arabidopsis, although immunolocalization of methylated cytosines showed comparable distributions and signal intensities in control and zebularine plants, in contrast to a marked reduction of the signal in *ddm1* mutants [19]. Thus, these data indicate cytosine methylation-independent mechanism(s) of the zebularine activity causes transcriptional activation of TEs and/or changes of chromocenters structure.

According to our data and that of others, genome hypomethylation correlates with telomere shortening in Arabidopsis [6,29,33]. Specifically, the loss of methylated cytosines in telomeric repeats was identified in *ddm1* and *met1* mutants, while Col plants germinated in the presence of hypomethylation drugs showed this decrease only in seedlings but not in leaves, despite the maintenance of the short telomere phenotype [6]. This corresponds with our observation that even the repeated exposure of Col and Ler seeds to zebularine during germination did not stabilize its effect on methylation of telomeric cytosines in plants which were further cultivated in the absence of the drug (Figure 5). On the other hand, hypomethylation of telomeric cytosines was maintained in Z/Z/Z Ws leaves, at similar levels in plants with long and short telomeres (Figure 5). The prolonged hypomethylation of telomeric cytosines may be related to a generally different response of Ws plants to the epigenetic stress. This was demonstrated by the delayed onset of telomere shortening in Ws plants (Figure 2c), despite the relevant hypomethylation of telomeric cytosines in 1G zebularine seedlings (Figure 5), and distinct behavior of Ws during cell culturing [28].

Telomeres and transposable elements represent functionally important genomic regions whose stability plays a key role in the maintenance of the genome integrity. Their specific responses to exposure to the epigenetically active drug zebularine and dynamics of the reversal of established changes demonstrate that plant cells dispose of efficient mechanisms to avoid deleterious effects of a critical telomere shortening and long-term transposon activation.

## 4. Materials and Methods

### 4.1. Cultivation of Plants

Seeds of *Arabidopsis thaliana* plants of Columbia-0 (Col), Landsberg erecta (Ler), and Wassilewskija (Ws) ecotypes were purchased from the NASC (Nottingham Arabidopsis Stock Centre, UK). Seeds were germinated for 7 days on half strength Murashige and Skoog (Duchefa, Biochemical, Haarlem, The Netherlands; M0255.0050) medium supplemented with 0.8% agar under short day conditions (8 h light, 100 mmol m^−2^s^−1^, 21 °C and 16 h dark, 19 °C). Plants were grown in the soil for 5 weeks under short day conditions favoring leaf growth, and then were cultivated under long day conditions (16 h light and 8 h dark) to support flowering. Leaves were harvested from 8-week-old plants. Using this protocol, O, Z/O, O/O, Z/Z/O, and O/O/O plants were cultivated (Figure 1).

Alternatively, seeds of Arabidopsis plants of Col, Ler, and Ws ecotypes were germinated for 7 days on agar plates with half strength Murashige and Skoog medium supplemented with 0.8% agar and 250 µM zebularine (Merck, Darmstadt, Germany). Seeds of one parental plant were taken to limit contribution of telomere lengths variability among representatives of respective ecotypes. Seedlings were transferred to the soil and plants were grown as described above. Using this protocol, Z, Z/Z, and Z/Z/Z plants were cultivated (Figure 1).

### 4.2. Analysis of Telomere Length by the Terminal Restriction Fragments (TRF) Method

This analysis was carried out according to the protocol described in [34]. Briefly, genomic DNA was isolated from leaves of 8-week-old plants by the protocol published in [35]; approximately 2 g (fresh weight) of leaves were collected from each plant. Five µg of genomic DNA were digested by the *Mse*I (NEB, Ipswich, MA, USA; recognition sequence T/TAA) and analyzed by Southern hybridization with a radioactively labeled telomeric probe synthesized by non-template PCR, using the modified protocol described in [36,37]. Hybridization signals corresponding to telomeric tracts (plus subtelomeres up to the first restriction site upstream of telomeres) were visualized using FLA7000 phosphoimager (FujiFilm, Minato, Japan) and analyzed by the MultiGauge V2.0 software (FujiFilm). The median of telomere length was calculated as ∑(OD_i_ × L_i_)/∑(OD_i_); OD_i_ is the signal intensity above background within the interval i, L_i_ is the molecular weight (kb) at the mid-point of the interval i.

### 4.3. Analysis of Transposable Elements Transcript Levels

RNA was isolated using the NucleoSpin RNA kit (Macherey-Nagel) from 7-day-old seedlings and leaves of 8-week-old plants; approximately 100 mg (fresh weight) of respective organ were collected for isolation, according to instructions in the isolation kit. One µg of RNA was reverse transcribed using M-MuLV reverse transcriptase (NEB, Ipswich, MA, USA) and random nonamers (Merck, Darmstadt, Germany) as primers. Relative levels of TEs transcripts were determined using quantitative PCR with EliZyme Green MIX AddROX (Elisabeth Pharmacon, Brno, Czech Republic) and Rotor-Gene 6000 (Qiagene, Hilden, Germany) cycler; sequences of primers are given in Supplementary Table S1. Signals of Athila2-I (At4g03790, Ty3/gypsy-like retrotransposon), SPM9 (At4g05505, CACTA-like transposon), and SPM11 (At4g06588, CACTA-like transposon) transcripts were related to the *ubiquitin10* reference transcript. Samples were analyzed in three technical replicates and data were evaluated by the ∆∆*C*t method [38]; for statistical analysis two sample F-test and t-test were used.

### 4.4. Analysis of Methylation of TE Cytosines

Genomic DNA was isolated from leaves of 8-week-old plants, as described in Section 4.2. The same protocol was applied for extraction of DNA from 1 g (fresh weight) of 7-day-old seedlings. Bisulfite conversion of 1 µg of genomic DNA was done using an EpiTect Bisulfite Kit (Qiagen, Hilden, Germany). During this process, non-methylated cytosines were converted to uraciles, while methylated cytosines remained intact [39]. The 334 bp fragment of the SPM11 transposon was amplified using primers enabling amplification of both converted and non-converted DNA (Supplementary Table S1) and MyTaq DNA polymerase (Meridian Bioscience, Memphis, TN, USA). PCR products were cloned (TOPO TA Cloning Kit; Thermo Fisher Scientific, Waltham, MA, USA) and sequenced (Macrogen, Seoul, South Korea). Methylation of 44 cytosines residing in an amplified region was evaluated using the CyMATE software [27]. Five to ten clones were analyzed per sample; main values of methylated cytosines in different sequence contexts and standard deviations were presented.

### 4.5. Analysis of Methylation of Telomeric Cytosines

Analysis was performed according to the protocol described in [7] with modifications presented in [20]. Genomic DNA from seedlings cultivated on three Petri dishes and from leaves of three plants grown from these seedling were isolated, as described in Section 4.2 and Section 4.4, respectively. For bisulfite conversion (described in Section 4.4), DNAs from respective organs were mixed in equimolar ratio. Alternatively, DNA isolated from seedlings taken from one Petri dish or leaves collected from one plant was treated by bisulfite. Then, 500 ng of DNA was transferred to the Hybond XL membrane (GE Healthcare, Chicago, IL, USA) and hybridized against radioactively labeled probes in ULTRAhybTM-Oligo Hybridization Buffer (Thermo Fisher Scientific, Waltham, MA, USA). The probe (TTAGRRT)_4_, R = A or G, was designed to hybridize with the fraction of telomeric repeats in which the inner cytosine was methylated and the other cytosines were either methylated or non-methylated; (ACCCTAA)_4_ probe hybridizing with the telomeric G-strand was used for DNA loading normalization. Hybridization signals were visualized using the FLA 7000 phosphoimager (FujiFilm) and evaluated using the MultiGauge software (FujiFilm).

## Supplementary Materials

Supplementary Materials can be found at https://www.mdpi.com/1422-0067/22/1/468/s1.

## Abbreviations

HR	Homologous recombination
TE	Transposable Eeement
TRF	Terminal restriction fragment
Col	Columbia-0
Ws	Wassilevskija
Ler	Landsberg erecta
