# Supplementary material for: Distinct Responses of Arabidopsis Telomeres and Transposable Elements to Zebularine Exposure
Source: Int J Mol Sci. 2021 Jan 5;22(1):468. doi: 10.3390/ijms22010468 (PMC7796508; doi:10.3390/ijms22010468)
Supplement: Supplementary file 1 [file ijms-22-00468-s001.pdf]

Table S1. Sequences of primers.

primer	sequence (5´→ 3´direction)	method
Athila 2-1Fw	GGGACATGCGGAATCTCTTG	analysis of transcription
Athila 2-1Rev	CTTCCACCGCTACAGGTTCG	analysis of transcription
SPM9Fw	GCCCGTGAGAATGATGAAGG	analysis of transcription
SPM9Rev	ATGCCTCTGCCTCACGATGT	analysis of transcription
SPM11Fw	GCGATGCCTTTTTGTGGAGA	analysis of transcription
SPM11Rev	GACCTAAGGGGACATGGTGGA	analysis of transcription
PAC1Fw	TCTCTTTGCAGGATGGGACAAGC	analysis of transcription
PAC1Rev	AGACTGAGCCGCCTGATTGTTTG	analysis of transcription
ubqRev	ACAAGATGAAGGGTGGAC	analysis of transcription
ubqFw	AACGGGAAAGACGATTAC	analysis of transcription
SPM11_BGSFw	CAGGYGTGTAAYGTTTGTGG	analysis of TE methylation
SPM11_BGSRev	TTRACCTCTTCTTCCACCRC	analysis of TE methylation

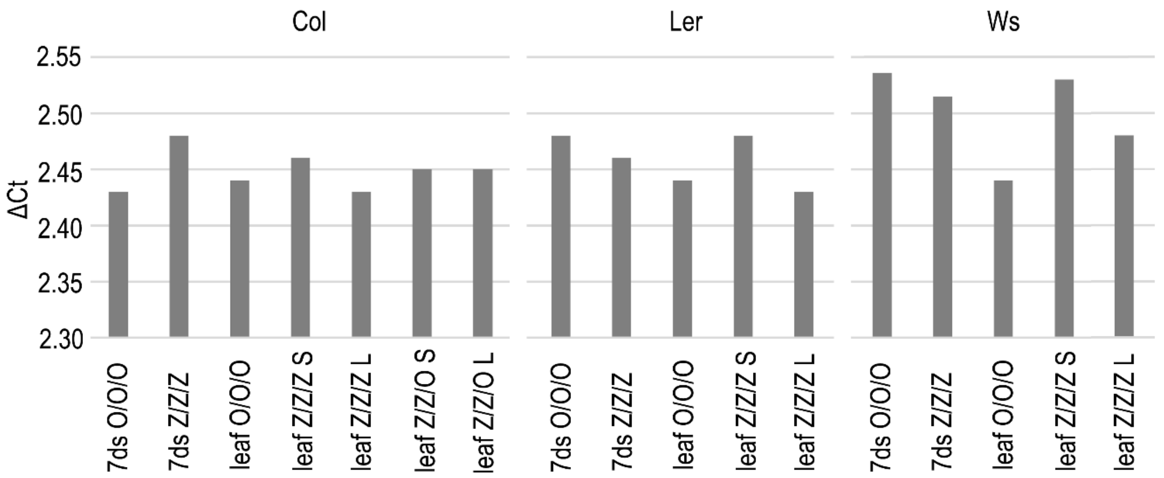

Figure S1. Levels of transcripts of the *PAC1* gene encoding constantly expressed protein. Ct numbers (number of the PCR cycle in which fluorescence increased significantly above the detection limit) were determined using Rotorgene6000 (Qiagene) software for *PAC 1* and *ubiquitine10* transcripts. ΔCt values as Ct(*pac1*) – Ct(*ubiquitin10*) were calculated; these values were comparable in all analyzed samples.

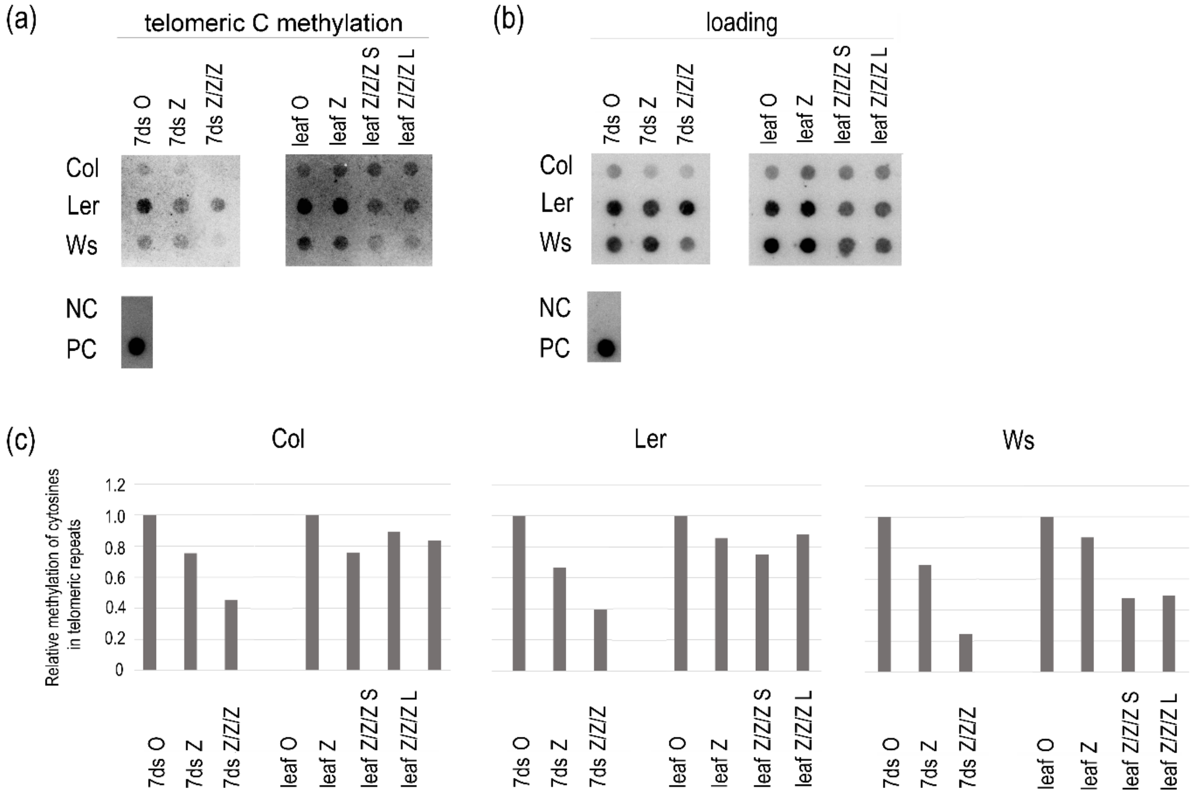

Figure S2. Relative levels of methylated cytosines in telomeric repeats in 7 days old seedlings (7ds) of *A. thaliana* plants germinated on control medium or exposed to 250 µM zebularine and in leaves of plants grown from these seedlings. DNA extracted from seedling grown on one Petri dish or leaf collected from one plant was converted by sodium bisulfite and hybridized with the oligonucleotide probe reflecting fraction of telomeres with methylated cytosines (a), and the probe complementary to the G-strand of telomeres to determine loading (b); the same membrane was sequentially hybridized with both probes. PC, positive control, genomic DNA from Col leaves non-converted by sodium bisulfite; NC, negative control, pUC19 plasmid DNA. (c) Relative methylation of cytosines in telomeric repeats. Intensities of hybridization signals in (a) and (b**)** were evaluated by the MultiGauge software (FujiFilm), and expressed as methylation/loading ratio. Signal ratios in respective control samples (7ds O, leaf O) were arbitrarily taken as 1. S, plants with short telomeres; L, plants with long telomeres (see Figure 2a). For experimental design and nomenclature of samples, see Figure 1.
